# Evaluation of newly developed wearable ear canal thermometer, mimicking the application to activities on sports and labor fields

**DOI:** 10.1186/s12576-023-00874-4

**Published:** 2023-07-18

**Authors:** Issei Kato, Hironori Watanabe, Kei Nagashima

**Affiliations:** 1 Graduate School of Human Sciences, Waseda University, Mikajima 2-579-15, Tokorozawa, Saitama 359-1192 Japan; 2 Japan Society for the Promotion of Science, Kojimachi 5-3-1, Chiyoda-ku, Tokyo, 102-0083 Japan; 3 Sustainable Energy and Environmental Society Open Innovation Research Organization, Waseda University, Nishiwaseda 1-6-1, Shinjuku-ku, Tokyo, 169-8050 Japan; 4 Body Temperature and Fluid Laboratory, Graduate School of Human Sciences, Faculty of Human Sciences, Waseda University, Mikajima 2-579-15, Tokorozawa, Saitama 359-1192 Japan

**Keywords:** Hyperthermia, Heat, Humidity, Fanning, Core body temperature

## Abstract

We evaluated the reliability of a newly developed wearable ear canal thermometer based on three different experiments, in which ear canal and rectal temperature (T_ear_ and T_rec_, respectively) were simultaneously monitored. In Experiment 1, participants sat at 28 °C and 50% relative humidity (RH), during which fanning or 41 °C lower legs water immersion was conducted. In Experiment 2, participants conducted a 70-min treadmill exercise (4 km/h, 0.5% slope) at 35 °C and 50% RH with intermittent fanning. In Experiment 3, participants completed a 20 min treadmill exercise (6 km/h, 5% slope) at 35 °C and 65% RH. Bland–Altman analysis for T_ear_ and T_rec_ showed the difference of − 0.2–0.3 °C and the limit of agreement of the mean ± 0.3–0.6 °C. The intraclass correlation coefficient was 0.44–0.83. The results may suggest that the ear canal thermometer is useful to assess core body temperature in sports and/or labor fields.

## Background

Maintenance of core body temperature is a goal of thermoregulation. Therefore, the measurement of core body temperature has been used to evaluate overall thermoregulatory responses. Core body temperature is regulated around 37 °C in healthy adult humans, although there are small inter- and intra-individual differences [1]. However, core body temperature deviates from the value of 37 °C for physiological and/or pathological reasons. Prolonged exercise and labor in heat are cases that increase core body temperature [2–5]. The extreme increase occasionally results in heat-related illness such as heat stroke. The increase in core temperature is determined by several factors such as the workload, environment, and individual thermoregulatory function. Therefore, it is difficult to estimate the risk of heat-related illness for each individual. We speculate that continuous measurement of core body temperature for individual can detect the risk in the earlier stage.

Continuous measurement of core body temperature is often conducted for clinical purposes such as surgery and insensitive care by placing thermometers in the core body such as the rectal [6], esophageal [7, 8], and bladder cavities, circulating blood in the pulmonary artery [9, 10], and the cerebral ventricle [11]. We also estimate core body temperature by placing a thermometer in the axilla, sublingual, and ear canal to know health conditions, although these body sites are not the ‘‘core body’’. For continuous monitoring of core body temperature in sports fields, measuring rectal and esophageal temperature is not practical because of the invasiveness and disturbing factor of exercise and physical labor. Sublingual and axillary temperature does not give us reliable data during exercise because the measurements are largely affected by ventilation and physical movement, respectively. Increased skin blood flow and sweating during exercise also affect skin temperature, which disturbs estimated core body temperature.

Tympanic temperature may be ideal for continuous assessment of core body temperature. It has been reported that tympanic temperature reflects esophageal temperature [12–15] as well as temperature of the hypothalamus, the center of the thermoregulation [16, 17]. The branch of the carotid artery, which reaches the brain, runs beside the tympanic membrane [18–21]. In addition, the tympanic membrane can be easily accessed from the ear canal. However, the classical measurement was conducted by placing a temperature sensor on the tympanic membrane, which carries a greater risk of damaging the membrane.

To reduce the risk of direct measurement of tympanic temperature, non-contact sensor devices have been developed [18, 22–25], which are placed as an earpiece-type thermometer. One general method is that, with the orifice of the ear canal closed, temperature in the ear canal is measured with ordinary temperature sensors such as thermocouple and thermistor, which is assumingly equilibrated with tympanic temperature [26]. This method may have following limitations: (i) there is no direct evidence showing that the value reflects tympanic temperature per se; (ii) hearing on one side is impaired; and (iii) time delay from the actual change of tympanic temperature is speculated. The other method is to assess temperature of the tympanic membrane and surrounding tissue by infrared thermometry [27–29]. This method may have an advantage in various sports and labor fields because it may reflect tympanic temperature more directly. However, the accuracy of the measurement is questioned due to the positioning of the sensor in the ear canal at the incorrect angle and/or incapacity of electromechanical and thermal properties of the sensor material [29]. Moreover, the environmental temperature may affect the measurement [30].

In the present study, we aimed to assess the reliability of the temperature obtained with a newly developed wearable earpiece-type infrared thermometer. For the assessment, the data were compared with rectal temperature, considered the gold standard for core body temperature. The comparison was conducted by four different experiments to know if the thermometer could address the concerns about the assessment of core body temperature with the infrared thermometer. In the experiments, fanning, lower-leg hot-water immersion, or treadmill exercise were conducted in different environments of normal or high ambient temperature with normal or high humidity.

## Methods

### *Data collection*

The analyses in the present study were conducted based on the data obtained from three different experimental studies in our laboratory, in which ear canal and rectal temperatures were continuously measured. The aims of the three studies were different from the present study. A total of 32 healthy volunteers participated in the studies. They were non-smokers and had no clinical history of cardiovascular, metabolic, or respiratory diseases. All the experimental procedures were approved by the Ethical Committee of Human Research, Waseda University (2020-097 and 2020-099) and we conducted the experiments following the Declaration of Helsinki (1983).

### *Experimental procedure*

**Fig. 1:**
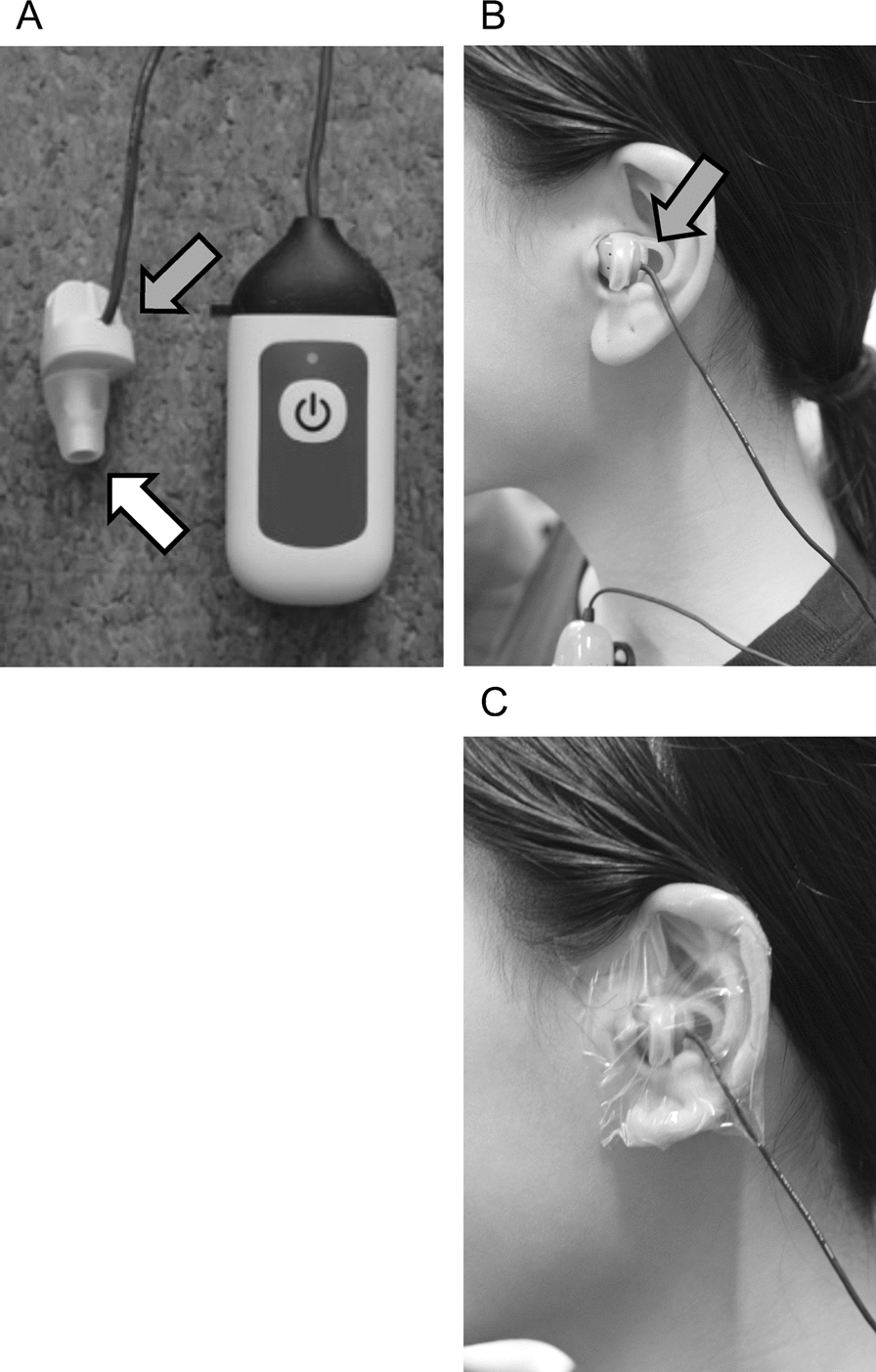
Photo-images of the wearable infrared ear canal thermometer used in the present study. The earpiece and microcomputer device (**A**), the image that thermometer is placed in the ear canal (**B**) and is covered with medical film (**C**). The white arrow denotes part of the earpiece placed in the ear canal. The grey arrow shows the tab of the earpiece, which is held between the orifice of the ear canal and the auricle

Briefly, participants had controlled diet and water intake (420 kcal breakfast with 500 ml water) for 3 h before each experiment. Participants wore a T-shirt, short pants, athletic shoes, and ankle-length socks. They rested for 30 min at least before each experiment, in a room where the ambient temperature was 28 ℃ with 50% relative humidity. During this period, all measurement devices were attached. They were then moved to an environmental chamber, the conditions of which are described in each experimental protocol. A thermistor probe for rectal temperature (T_rec_) measurement (401 J, Nikkiso-Thermo, Musashino, Japan) was placed, of which tip was located at 13 cm from the anus. A wearable thermometer (VTB01, Vitarate, Tokyo, Japan) was placed in the ear canal. The thermometer consisted of an earpiece wired to a microcomputer device (Fig. 1). The earpiece had a two thin-film infrared sensor inside facing to the tympanic membrane, which was used in another thermometer for medical use [31]. The infrared sensor was designed to detect the net heat flow from the tympanic membrane and surrounding tissues to the outside environment. The earpiece shape and data collection methods were newly developed. Figure 1A shows the earpiece connected to the thermometer controller unit. One part of the earpiece (indicated by a white arrow) was placed in the ear canal. The other part, with a small tab (indicated by a gray arrow), was held between the orifice of the ear canal and the auricle (Fig. 1B). The earpiece did not occlude the ear canal, but held its position even during physical activity.

The probe for T_rec_ was connected to a data logger (N543, Nikkiso-Thermo, Musashino, Japan), and the temperature was recorded every 30 s. The temperature data with the ear canal thermometer (T_ear_) were collected every 30 s, via Bluetooth, with an application installed on a smartphone (iPhone 11, Apple, Cupertino, USA), which was visible on the screen and recorded on local and cloud recorders. The data were stored as a file of comma-separated values and analyzed later. The ear was covered with medical film (Tegaderm, 3 M Company, Saint Paul, USA) to reduce the direct influence of ambient ventilation (Fig. 1C). The data of T_rec_ and T_ear_ were obtained every 30 s throughout the experiments.

In Experiment 1, nine participants (six males and three females; age, 23 ± 2 years; and body weight, 61.0 ± 9.8 kg) conducted two different trials separated by at least 2 days. Participants sat on a chair for 120 min, during which fanning (fanning trial) or 41 °C water immersion of both lower legs (water immersion trial) was conducted at 30–90 min from the onset of the experiment. In fanning trial, the wind was applied to the participants with a fan placed 200 cm from the participants and at 80 cm height (35 cm diameter, MF-FR35D, U-ING, Osaka, Japan) with a wind speed of 2 m/s at the participants’ torso level. The environmental condition in both trials was T_a_ of 28 °C and 50% RH.

In Experiment 2, 11 male participants (age, 22 ± 1 years; and body weight, 65.9 ± 8.8 kg) rested in a sitting position for 50 min, followed by 70 min treadmill exercise at 4 km/h with 0.5% slope. During the experiment, fanning was given to the torsos of participants for 10 min five times starting at 10, 30, 60, 80, and 100 min from the onset of the experiment. The setting of fanning was the same as that of Experiment 1*.* The environmental condition was T_a_ of 35 °C and 50% RH.

In Experiment 3, nine participants (five males and four females; age, 23 ± 3 years; and body weight, 64.3 ± 12.4 kg) ran on a treadmill (TRM731, Precor, Woodinville, USA) at 6 km/h with 5% slope for 20 min after 10 min standing rest. The environmental condition was T_a_ of 35 °C and 65% RH.

### *Statistical analysis*

R was used for statistical analyses [32, 33]. The data for T_rec_ and T_ear_ were averaged every 5 min. The Kolmogorov–Smirnov test verified the normality of the data, and Levene’s test clarified the homogeneity of variance. Differences between T_rec_ and T_ear_ were evaluated by the two-way analysis of variance for repeated measurement in each experiment (time and temperature factors). A post hoc test was conducted using the Bonferroni method when a significant main effect or interaction was observed.

To assess the agreement between T_rec_ and T_ear_, Bland–Altman analysis [34] was conducted. Differences between T_rec_ and T_ear_ for the mean of T_rec_ and T_ear_ were plotted in each datum (Bland–Altman plot). The Pearson correlation coefficient and the 95% limit of agreement (LoA; means ± 1.96 SD) were calculated. The intraclass correlation coefficient (ICC; two-way random-effects model, absolute agreement) between T_rec_ and T_ear_ was also calculated based on the reported guideline [35]. All data are presented as the mean ± SD. The null hypothesis was rejected at *P* < 0.05.

## Results

### *Experiment 1: fanning trial; Trec and Tear during rest, rest with fanning, and recovery in a normal ambient humidity condition*

**Fig. 2:**
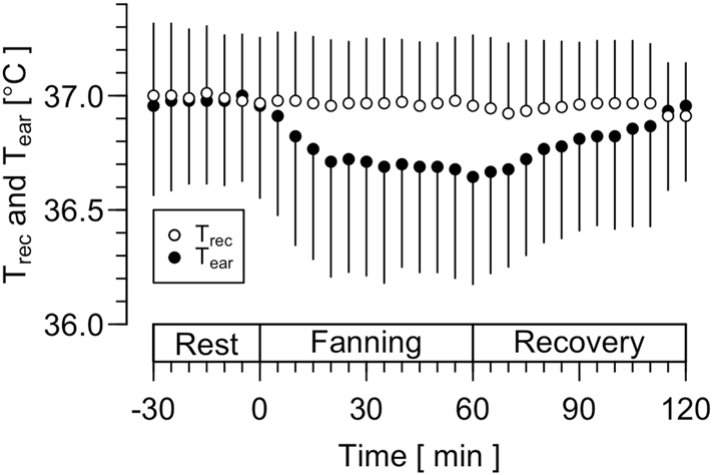
Rectal (T_rec_) and ear canal temperature (T_ear_) during sitting rest with fanning at 0 – 60 min in a normal environment (fanning trial in Experiment 1). Values are as means ± SD

Figure 2 shows T_rec_ and T_ear_, including during 30-min rest, 60-min rest with continuous fanning, and the recovery. Main effect was observed in the temperature factor (*F*_(1, 554)_ = 25.097, *P* < 0*.*001), but not in the time factor (*F*_(1, 554)_ = 3.441, *P* = 0.0641). There was no interaction between the two factors (*F*_(1, 554)_ = 0.511, *P* = 0.4751). No significant differences were observed between T_rec_ and T_ear_ (*P* > 0.116).

**Fig. 3:**
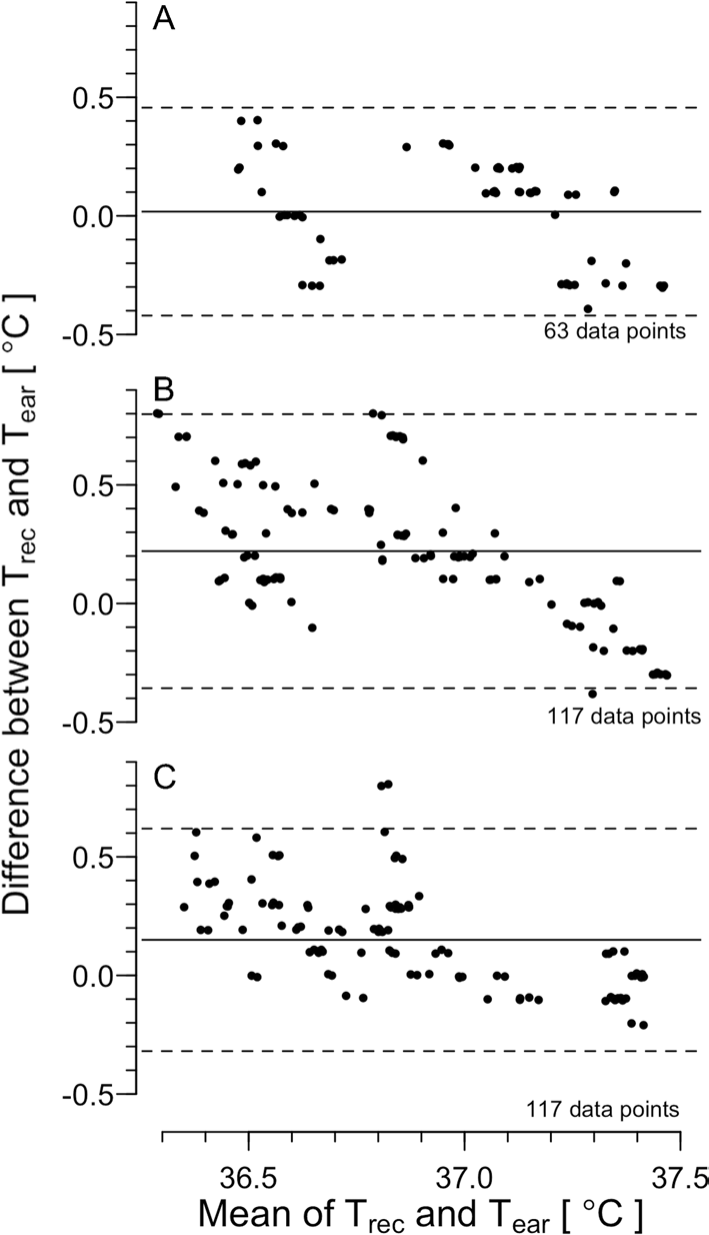
The Bland–Altman plot for rectal (T_rec_) and ear canal temperature (T_ear_) during rest (**A)**, fanning (**B)**, and recovery periods (**C)** in the fanning trial in Experiment 1. The plots show (T_ear_–T_rec_) against mean of T_ear_ and T_rec_. Each datum denotes that of each time point in each participant. Solid and dashed lines indicate mean of T_rec_ and T_ear_ (mean) and 95% limit of agreement (LoA, Mean ± 1.96 SD), respectively. The number of the data points is also presented

Figure 3A, B, and C shows the Bland–Altman plot for T_rec_ and T_ear_ in the rest, rest with fanning, and the recovery periods, respectively. The correlation coefficient for the difference in T_rec_ and T_ear_ and mean of T_rec_ and T_ear_ was significant in the three periods (− 0.36, *P* = 0.003; − 0.66, *P* < 0.001, and − 0.57, *P* < 0.001; respectively). In the rest period, LoA was 0.0 ± 0.4 °C, and ICC was 0.75 (*P* < 0.001). In the rest period with fanning, LoA was 0.2 ± 0.6 °C, and ICC was 0.58 (*P* < 0.001). In the recovery, LoA was 0.2 ± 0.5 °C, and ICC was 0.68 (*P* < 0.001).

### *Experiment 1: water immersion trial; T*_ear_*and T*_rec_*during rest, rest with 41 °C lower legs water immersion and the recovery in a normal ambient humidity condition*

**Fig. 4:**
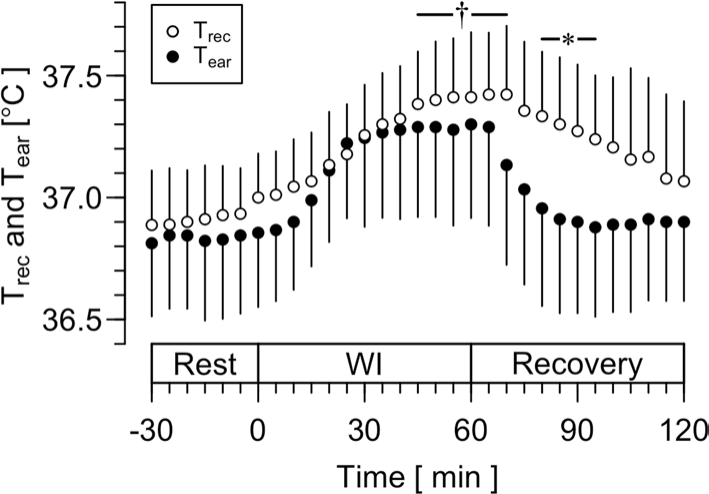
Rectal (T_rec_) and ear canal temperature (T_ear_) during sitting rest, 41 °C water immersion of both lower legs, and the recovery in a normal environment (water immersion trial in Experiment 1). Values are means ± SD. *, Significant difference between T_rec_ and T_ear_, *P* < 0.05. †, Significant difference from the value of T_rec_ at 0 min, *P* < 0.05. WI, water immersion

Figure 4 shows T_rec_ and T_ear_ during 30 min rest, 60 min rest with lower-leg hot-water immersion, and the recovery. A significant main effect was observed for both time (*F*_(1, 552)_ = 21.532, *P* < 0.001) and temperature (*F*_(1, 552)_ = 32.636, *P* < 0.001). There was a significant interaction between these two factors (*F*_(1, 552)_ = 8.707, *P* = 0.0033). T_rec_ was significantly different from the value at 30 min at 70–100 min (*P* < 0.05) and was greater than T_ear_ at 110–125 min (*P* < 0.05).

**Fig. 5:**
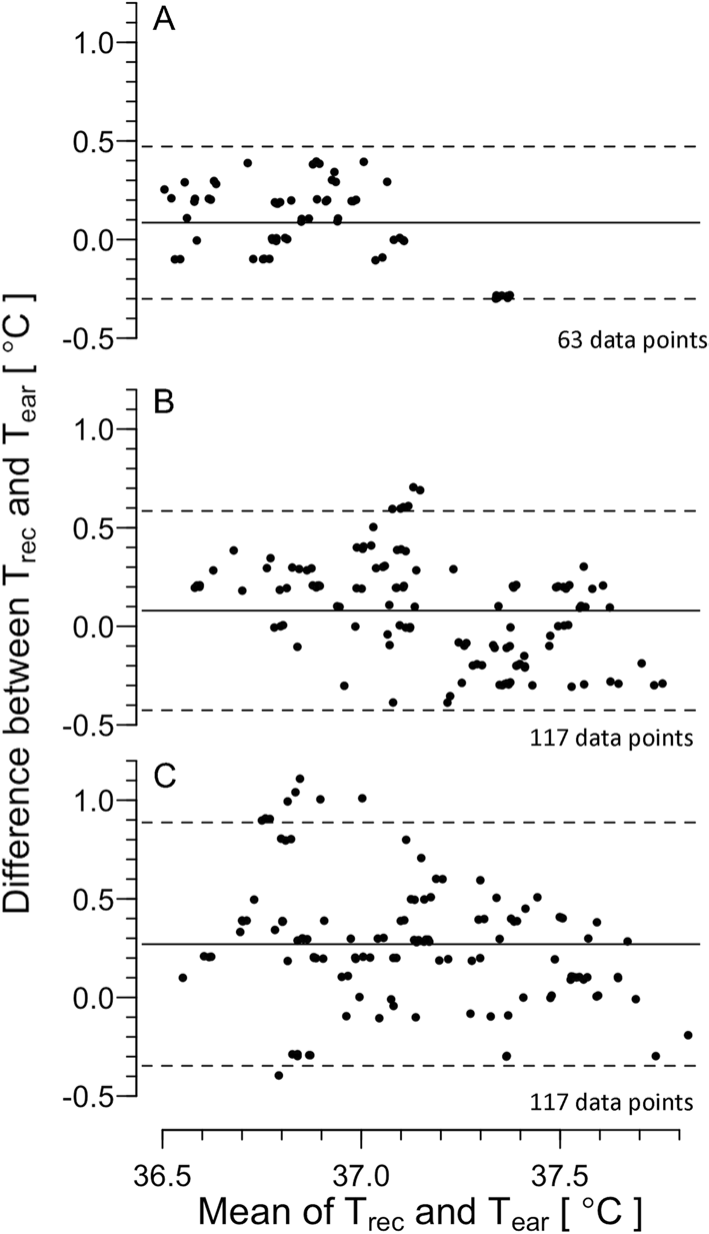
The Bland–Altman plot for T_rec_ and T_ear_ during rest (**A)**, the water immersion (**B)**, and the recovery periods (**C)** in the water immersion trial in Experiment 1. Solid and dashed lines indicate mean of T_rec_ and T_ear_ (mean) and 95% limit of agreement (LoA, mean ± 1.96 SD), respectively. The number of the data points is also presented

Figure 5A, B, and C shows the Bland–Altman plot in the rest, rest with the water immersion and recovery periods, respectively. The correlation coefficient was significant in the three periods (− 0.50, *P* < 0.001;− 0.44, *P* < 0.001; and − 0.26, *P* = 0.03; respectively). In the rest, LoA was 0.1 ± 0.4 °C, and ICC was 0.64 (*P* < 0.001). In the rest with the water immersion, LoA was 0.1 ± 0.5 °C, and ICC was 0.63 (*P* < 0.001). In the recovery, LoA was 0.3 ± 0.6 °C, and ICC was 0.44 (*P* = 0.01).

### *Experiment 2: T*_*rec*_* and T*_*ear*_* during rest and treadmill exercise with intermittent fanning in a hot and normal humidity condition*

**Fig. 6:**
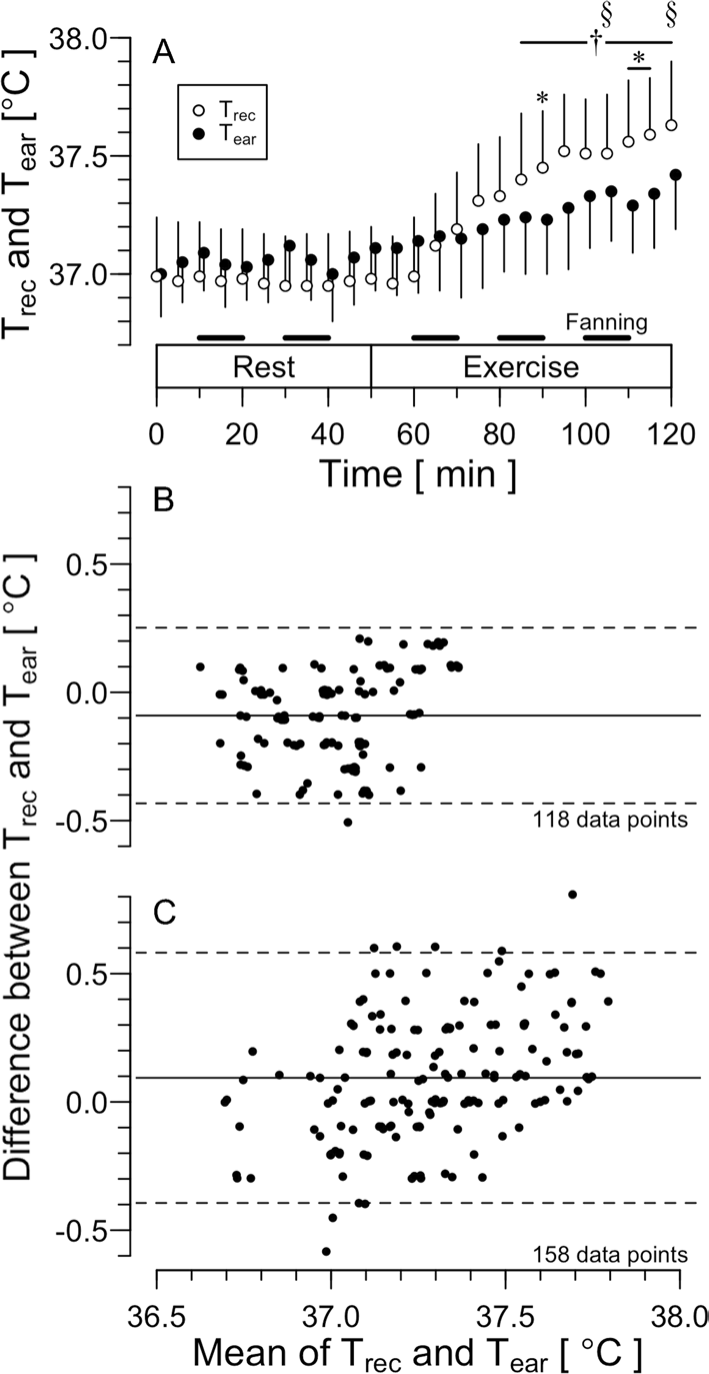
Rectal (T_rec_) and ear canal temperature (T_ear_) during standing rest and treadmill exercise with intermittent fanning (**A)** in a hot environment and the Bland–Altman plot for the resting (**B)** and exercise periods (**C)**. Values are means ± SD. Black bars in **A** denote the period during which fanning was applied. *, Significant difference between T_rec_ and T_ear_, *P* < 0.05. †, Significant difference from time 0 min in T_rec_, *P* < 0.05. §, Significant difference from time 0 min in T_ear_, *P* < 0.005. Solid and dashed lines in B and C indicate Mean and 95% LoA, respectively. The number of the data points is also presented

Figure 6A illustrates T_rec_ and T_ear_ in the rest (0–50 min) and exercise periods (50–120 min). T_rec_ and T_ear_ remained unchanged without any differences during the 50-min rest (37.0 ± 0.2 °C and 37.0 ± 0.1 °C, respectively). There was a significant main effect of time (*F*_(1, 526)_ = 287.479, *P* < 0.001), but not of temperature (*F*_(1, 526)_ = 1.200, *P* = 0.273). A significant interaction was observed between these two factors (*F*_(1, 526)_ = 36.380, *P* < 0.001). At 85–120 min, T_rec_ became higher than the level at 0 min (*P* < 0.05). T_ear_ increased at 105 and 120 min (*P* < 0.05). T_ear_ was lower than T_rec_ at 90 and 110–115 min (*P* < 0.05).

Figure 6B and C shows the Bland–Altman plot in the rest and treadmill exercise periods, respectively. The correlation coefficient was significant in both resting and exercise periods (0.20, *P* = 0.02; and 0.41, *P* < 0.01, respectively). In the resting period, LoA was − 0.1 ± 0.3 °C, and ICC was 0.54 (*P* < 0.001). In the exercise period, LoA was 0.1 ± 0.5 °C, and ICC was 0.58 (*P* < 0.001).

### *Experiment 3: T*_*rec*_* and T*_*ear*_* during treadmill exercise in a hot and humid condition*

**Fig. 7:**
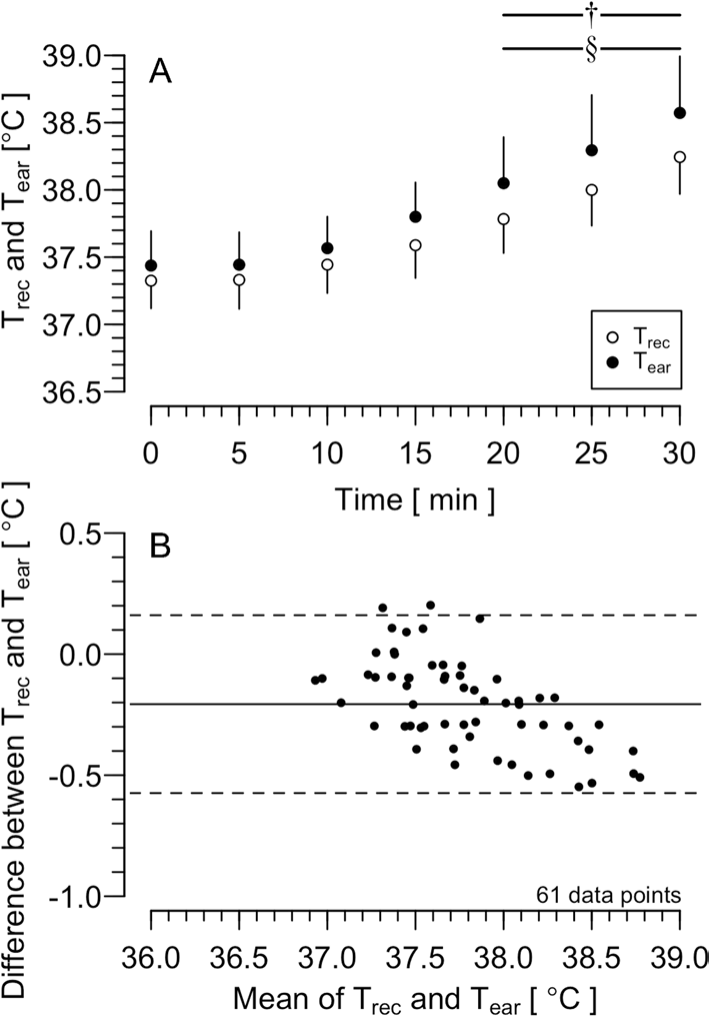
Rectal (T_rec_) and ear canal temperature (T_ear_) during treadmill exercise in a hot and humid environment (**A)** and the Bland–Altman plot for T_rec_ and T_ear_ (**B)**. Values are means ± SD. †, Significant difference from the value at 0 min in T_rec_, *P* < 0.005. Significant difference from the value at 0 min in T_ear_. §, significant difference between T_rec_ and T_ear_, *P* < 0.01. Solid and dashed lines indicate mean of T_rec_ and T_ear_ (mean) and 95% limit of agreement (LoA, mean ± 1.96 SD), respectively. The number of the data points is also presented. Two data points were excluded because of the measurement noise for T_rec_ (61 in total)

Figure 7A illustrates T_rec_ and T_ear._ T_rec_ and T_ear_ remained unchanged without any differences during the 10-min rest (37.2 ± 0.5 °C and 37.3 ± 0.3 °C at 0 min, respectively). Significant main effects were observed for both time and temperature (*F*_(1, 118)_ = 193.756, *P* < 0.001 and *F*_(1, 118)_ = 16.730, *P* < 0.001, respectively). There was no significant interaction between these two factors (*F*_(1, 118)_ = 2.559, *P* = 0.112). Both T_rec_ and T_ear_ increased from the 0-min values at 20–30 min (*P* < 0.005; 38.2 ± 0.4 °C and 38.5 ± 0.2 °C at 30 min), respectively. There were no significant differences between T_rec_ and T_ear_ during exercise (*P* > 0.13).

Figure 7B shows the Bland–Altman plot during the treadmill exercise in a hot and humid environment. The correlation coefficient between T_rec_ and T_ear_ was significant (− 0.60, *P* < 0.001). The LoA was − 0.2 ± 0.4 °C, and ICC was 0.83 (*P* = 0.007). Two data points were excluded because of the measurement noise for T_rec_.

Table 1 summarized the analyses of the Bland–Altman plot and ICC in Experiments 1, 2, and 3.

**Table 1:** Analysis based on the Bland–Altman plot for rectal temperature and ear canal temperature in Experiments 1, 2, and 3

Experiment	Period	Correlation coefficients (*P* value)	LoA	ICC (*P* value)
Experiment 1, fanning trial	Rest	− 0.36 (*P* = 0.003)	0.0 ± 0.4 ℃	0.75 (*P* < 0.001)
	Fanning	− 0.66 (*P* < 0.001)	0.2 ± 0.6 ℃	0.58 (*P* < 0.001)
	Recovery	− 0.57 (*P* < 0.001)	0.1 ± 0.5 ℃	0.68 (*P* < 0.001)
Experiment 1, water immersion trial	Rest	− 0.50 (*P* < 0.001)	0.1 ± 0.4 ℃	0.64 (*P* < 0.001)
	Water immersion	− 0.44 (*P* < 0.001)	0.1 ± 0.5 ℃	0.63 (*P* < 0.001)
	Recovery	− 0.26 (*P* = 0.03)	0.3 ± 0.6 ℃	0.44 (*P* = 0.01)
Experiment 2	Rest with intermittent fanning	0.24 (*P* = 0.006)	− 0.1 ± 0.3 ℃	0.54 (*P* < 0.001)
	Exercise with intermittent fanning	0.39 (*P* < 0.001)	0.1 ± 0.5 ℃	0.58 (*P* < 0.001)
Experiment 3	Exercise	− 0.60 (*P* < 0.001)	− 0.2 ± 0.4 ℃	0.83 (*P* = 0.007)

*LoA* limits of agreement, *ICC* intraclass correlation coefficient

## Discussion

In the present study, we evaluated the reliability of a newly developed earpiece-type infrared thermometer, assuming that the thermometer is used for the safety during sports activities and physical labor. Therefore, as the first step, we compared the changes in rectal temperature and ear canal temperature that were obtained with the thermometer, during thermal and environmental stimuli mimicking the activities in the fields.

During sitting rest in a normal ambient and humidity condition before applying fanning and water immersion (*T*_a_ = 25 °C and 50% relative humidity; the first 30-min rest period in Experiment 1), the LoA was 0.0 ± 0.4 °C and 0.1 ± 0.4 °C, respectively, with ICC of high agreement (Figs. 3, 5A and Table 1). Some studies have reported that, when compared core body temperature obtained among different core body sites, the LoA was reported as mean ± 0.3 °C [36–38]. In addition, a direct measurement of intra-gastrointestinal temperature using an ingestible pill-type thermometer, the obtained temperature fluctuated by ± 0.3 °C [39]. We also found such fluctuation of ± 0.1 °C during the measurement of T_rec_. Therefore, we assume that the value of LoA is fully acceptable. In addition, the small mean difference with ICC of high agreement may suggest that the ear canal temperature obtained with the present thermometer can be reliable to estimate core body temperature during sitting rest in a normal environment, where core body temperature is stable.

When heat was applied to the core body by the lower-leg water immersion (the water immersion period in the water immersion trial in Experiment 1), we found statistical increase in T_rec_ during the water immersion but not in T_ear_ (Fig. 4). In addition, T_rec_ was greater than T_ear_ during the recovery period. However, as observed in Fig. 4, it appears that T_ear_ rapidly changed during the water immersion and recovery, compared with T_rec_. One possible explanation for this difference could be regional variations among the body sites where core body temperature was measured. It is plausible that the heat applied to the lower legs caused a greater increase in the temperature of venous blood passing near the rectum and the surrounding pelvic tissues. On the other hand, T_ear_ may reflect the temperature of circulating blood, which is lower than that of venous blood and the tissues in the pelvis. The response of T_ear_ to changes in blood temperature may be faster than that of T_rec_ because T_rec_ was assessed in the rectum, which is separated from the blood vessels by a thicker wall and surrounding tissues. However, the LoA was 0.1 ± 0.5 °C with ICC of high agreement (Fig. 5B and Table 1). Although an increase in the LoA was observed, the results may show that T_ear_ reflects the increase in core body temperature well.

Nadel and Horvath [30] reported that tympanic temperature assessed by direct measurement fluctuates both in heat and cold: higher than the rectal temperature in heat and lower in cold. They speculated that changes in skin temperature alter blood supply to the tympanic membrane, resulting in an influence of ambient temperature on tympanic temperature. They also suggested that the convection in the ear canal does not affect tympanic temperature. However, Coso et al. [40] reported that, when ear canal temperature was assessed by infrared thermometry in a hot environment (T_a_ of 37 °C and 27% humidity) with airflows of 2.5 m/s, the ear canal temperature became lower than rectal temperature and showed lower LoA− 1.0 ± 1.1 °C. Based on this report, we used medical film to cover the ear canal in all experiments in the present study (Fig. 1C). We did not find any statistical differences between T_rec_ and T_ear_ during fanning in Experiment 1 (Fig. 2). The LoA was 0.2 ± 0.6 °C despite ICC of moderate agreement (Fig. 5B and Table 1). In addition, when intermittent fanning was applied to resting participants in heat (rest period in Experiment 2), the LoA was − 0.1 ± 0.3 °C with an ICC of moderate agreement. These results indicated the reliability of the ear canal thermometer. We found a main effect of the temperature in the fanning trial in Experiment 1, which may suggest lower T_ear_ than T_rec_ as visually assessed in Fig. 2. However, since there was no interaction between the temperature and time effects, we cannot determine if T_ear_ decreased due to the influence of the fanning. The change in the mean of the LoA during the fanning period may indicate the influence of the fanning (Fig. 3). One possible reason for the change of T_ear_ may be a change in the facial skin temperature. Therefore, caution should be exercised when there are rapid changes in facial skin temperature.

During treadmill exercise in heat with intermittent fanning (exercise period in Experiment 2), T_ear_ was lower than T_rec_. However, T_ear_ reflects T_rec_ well, showing a LoA of − 0.1 ± 0.5 °C with ICC of moderate agreement. When treadmill exercise was conducted in a hot and humid condition (Experiment 3), a significant main effect of temperature was observed. As speculated in the water immersion trial in Experiment 1, T_ear_ may have reflected the increase in the temperature of circulating blood faster than T_rec_. The ICC showed a good agreement, and mean differences remained small (0.3 °C; Fig. 7B).

The ear canal temperature was measured to assess the tympanic temperature. The importance of the tympanic temperature as an index of core body temperature has been reported previously [12–17]. When measuring the tympanic temperature using infrared thermometry, the capacity of the sensor material must be considered [29]. In addition, the position of the infrared sensor in the ear canal is a key factor in the measurement because radiation from the tympanic membrane must reach the sensor face at a right angle. Daanen [24] reported that when the tympanic temperature is assessed using infrared thermometry, the morphology of the ear canal can result in erroneous measurements, affecting the sensor's position. Based on the present study, we conclude that the proposed infrared thermometer decreases some potential errors when evaluating core body temperature using an ear canal thermometer [41]. The first reason for this achievement is the design of the earpiece, which maintains the positioning of the sensor in the ear canal at the correct angle to the tympanic membrane. The second reason is that environmental conditions slightly influence the sensor material.

In all experimental data, we found a significant correlation coefficient for the differences in T_rec_ and T_ear_ and the means in the Bland–Altman analyses, suggesting systematic errors. As discussed above, the LoAs in Experiments 1–3 were sufficiently small (Table 1). Therefore, within the range of changes in T_ear_ in the present study conditions, we believe that systematic errors do not have to be considered. However, when a greater increase in the core body temperature is expected, such as during prolonged labor and/or exercise with a higher workload in hot and humid conditions, it may not show the true core body temperature. We plan to apply the present ear canal thermometer to labor and/or sports fields in the future. Experiment 3 mimicked actual labor and exercise in hot and humid conditions, where the risk of heat-related diseases was greater. T_rec_ reached about 38.5 ℃ at the end of the 20 min exercise, which may not be considered a physiological condition inducing heat-related illness in most individuals. Further studies under extreme conditions are required. In addition, we used medical film to cover the orifice of the ear canal to reduce the influence of wind. The use of medical film may be a limiting factor in labor and/or the sports field, requiring further studies to assess its influence without the medical film. However, using the present thermometer, we could detect abnormal increase in core body temperature in the earlier stage. For example, an abnormal increase is speculated to be due to differences from other individuals in the same field and/or from the data of one’s own core body temperature on different days.

## Conclusion

In this study, we assessed the reliability of the newly developed infrared ear canal thermometer. Under fanning, core body heating, and exercise conditions, which may disturb the data obtained using an infrared thermometer, the comparison between the ear canal temperature and rectal temperature was conducted. The difference of the two values was found during fanning and exercise. However, based on the Bland–Altman analyses, we demonstrated the reliability of the T_ear_ for the estimation of core body temperature (T_rec_ in the present study). The present study suggests that continuous measurement of T_ear_ with the present thermometer could be a tool for decreasing heat-related diseases in labor and/or sports fields. We do not know whether the thermometer is applicable to individuals at higher risk, such as older adults and those with diabetes and dehydration. Therefore, further evaluations are required.

## Data Availability

The datasets used and/or analyzed during the current study are available from the corresponding author upon reasonable request.

## Abbreviations

T_a_: Ambient temperature
RH: Relative humidity
T_rec_: Rectal temperature
T_ear_: Ear canal temperature
LoA: Limits of agreement
ICC: Intraclass correlation coefficient
HW: Hot-water immersion

## References

[1] Mackowiak PA, Wasserman SS, Levine MM (1992). A critical appraisal of 98.6°f, the upper limit of the normal body temperature, and other legacies of carl reinhold August Wunderlich. JAMA.

[2] Drust B, Rasmussen P, Mohr M (2005). Elevations in core and muscle temperature impairs repeated sprint performance. Acta Physiol Scand.

[3] Kenny GP, Webb P, Ducharme MB (2008). Calorimetric measurement of postexercise net heat loss and residual body heat storage. Med Sci Sports Exerc.

[4] Kenny GP, Dorman LE, Webb P (2009). Heat balance and cumulative heat storage during intermittent bouts of exercise. Med Sci Sports Exerc.

[5] Todd G, Butler JE, Taylor JL, Gandevia SC (2005). Hyperthermia: a failure of the motor cortex and the muscle. J Physiol.

[6] Mead J, Bonmarito CL (1949). Reliability of rectal temperatures as an index of internal body temperature. J Appl Physiol.

[7] Shiraki K, Konda N, Sagawa S (1986). Esophageal and tympanic temperature responses to core blood temperature changes during hyperthermia. J Appl Physiol.

[8] Molnar GW, Read RC (1974). Studies during open-heart surgery on the special characteristics of rectal temperature. J Appl Physiol.

[9] Shellock FG, Rubin SA (1982). Simplified and highly accurate core temperature measurements. Med Prog Technol.

[10] Furlong D, Carroll DL, Finn C (2015). Comparison of temporal to pulmonary artery temperature in febrile patients. Dimens Crit Care Nurs.

[11] Childs C, Machin G (2009). Reliability issues in human brain temperature measurement. Crit Care.

[12] Cabanac M, Germain M, Brinnel H (1987). Tympanic temperatures during hemiface cooling. Europ J Appl Physiol.

[13] Brinnel H, Cabanac M (1989). Tympanic temperature is a core temperature in humans. J Therm Biol.

[14] Childs C, Harrison R, Hodkinson C (1999). Tympanic membrane temperature as a measure of core temperature. Arch Dis Child.

[15] Moran JL, Peter JV, Solomon PJ (2007). Tympanic temperature measurements: are they reliable in the critically ill? A clinical study of measures of agreement*. Crit Care Med.

[16] Benzinger TH (1959). On physical heat regulation and the sense of temperature in man. Proc Natl Acad Sci USA.

[17] Mariak Z, Lewko J, Luczaj J (1994). The relationship between directly measured human cerebral and tympanic temperatures during changes in brain temperatures. Eur J Appl Physiol.

[18] Farnell S, Maxwell L, Tan S (2005). Temperature measurement: comparison of non-invasive methods used in adult critical care. J Clin Nurs.

[19] Khorshid L, Eşer İ, Zaybak A, Yapucu Ü (2005). Comparing mercury-in-glass, tympanic and disposable thermometers in measuring body temperature in healthy young people. J Clin Nurs.

[20] Hoffman C, Boyd M, Briere B (1999). Evaluation of three brands of tympanic thermometer. Can J Nursing Res Archiv.

[21] Giuliano K, Giuliano A, Scott S (2000). Temperature measurement in critically ill adults: a comparison of tympanic and oral methods. Am J Crit Care Off Publ Am Assoc Crit-Care Nurses.

[22] Shibasaki M, Kondo N, Tominaga H (1998). Continuous measurement of tympanic temperature with a new infrared method using an optical fiber. J Appl Physiol.

[23] Gasim GI, Musa IR, Abdien MT, Adam I (2013). Accuracy of tympanic temperature measurement using an infrared tympanic membrane thermometer. BMC Res Notes.

[24] Daanen HAM (2006). Infrared tympanic temperature and ear canal morphology. J Med Eng Technol.

[25] Onur OE, Guneysel O, Akoglu H (2008). Oral, axillary, and tympanic temperature measurements in older and younger adults with or without fever. Eur J Emerg Med.

[26] Roossien CC, Heus R, Reneman MF, Verkerke GJ (2020). Monitoring core temperature of firefighters to validate a wearable non-invasive core thermometer in different types of protective clothing: concurrent in-vivo validation. Appl Ergonom.

[27] Ota H, Chao M, Gao Y (2017). 3D printed “earable” smart devices for real-time detection of core body temperature. ACS Sens.

[28] Matsumoto K, Temiz Y, Taghavi H, et al. An earbud-type wearable (A hearable) with vital parameter sensors for early detection and prevention of heat-stroke. In: 2019 41st Annual International Conference of the IEEE Engineering in Medicine and Biology Society (EMBC). pp 7049–7055. 2019. 10.1109/EMBC.2019.8856821 31947461

[29] Chaglla EJS, Celik N, Balachandran W (2018). Measurement of core body temperature using graphene-inked infrared thermopile sensor. Sensors.

[30] Nadel ER, Horvath SM (1970). Comparison of tympanic membrane and deep body temperatures in man. Life Sci.

[31] Kiya T, Yamakage M, Hayase T (2007). The usefulness of an earphone-type infrared tympanic thermometer for intraoperative core temperature monitoring. Anesth Analg.

[32] R Core Team. R: A language and environment for statistical computing. 2021.

[33] Ihaka R, Gentleman R (1996). R: a language for data analysis and graphics. J Comput Graph Stat.

[34] Martin Bland J, Altman DG (1986). Statistical methods for assessing agreement between two methods of clinical measurement. Lancet.

[35] Koo TK, Li MY (2016). A guideline of selecting and reporting intraclass correlation coefficients for reliability research. J Chiropr Med.

[36] Notley SR, Meade RD, Kenny GP (2021). Time following ingestion does not influence the validity of telemetry pill measurements of core temperature during exercise-heat stress: the journal temperature toolbox. Temperature.

[37] Tokizawa K, Shimuta T, Tsuchimoto H (2022). Validity of a wearable core temperature estimation system in heat using patch-type sensors on the chest. J Ther Biol.

[38] Tsadok I, Scheinowitz M, Shpitzer SA (2021). Assessing rectal temperature with a novel non-invasive sensor. J Ther Biol.

[39] Kolka MA, Quigley MD, Blanchard LA (1993). Validation of a temperature telemetry system during moderate and strenuous exercise. J Therm Biol.

[40] Coso JD, Aguado-Jimenez R, Mora-Rodriguez R (2008). Infrared tympanic thermometry in a hot environment. Int J Sports Med.

[41] Ainsworth BE, Haskell WL, Whitt MC (2000). Compendium of physical activities: an update of activity cod medicine & science in sports & exercise. Med Sci Sports Exerc.

[42] Huggins R, Glaviano N, Negishi N (2012). Comparison of rectal and aural core body temperature thermometry in hyperthermic, exercising individuals: a meta-analysis. J Athl Train.

